# Recovering Protein-Protein and Domain-Domain Interactions from Aggregation of IP-MS Proteomics of Coregulator Complexes

**DOI:** 10.1371/journal.pcbi.1002319

**Published:** 2011-12-29

**Authors:** Amin R. Mazloom, Ruth Dannenfelser, Neil R. Clark, Arsen V. Grigoryan, Kathryn M. Linder, Timothy J. Cardozo, Julia C. Bond, Aislyn D. W. Boran, Ravi Iyengar, Anna Malovannaya, Rainer B. Lanz, Avi Ma'ayan

**Affiliations:** 1 Department of Pharmacology and Systems Therapeutics, Systems Biology Center New York (SBCNY), Mount Sinai School of Medicine, New York, New York, United States of America; 2 Department of Pharmacology, New York University School of Medicine, New York, New York, United States of America; 3 Department of Molecular and Cellular Biology, Baylor College of Medicine, Houston, Texas, United States of America; University of Zurich and Swiss Institute of Bioinformatics, Switzerland

## Abstract

Coregulator proteins (CoRegs) are part of multi-protein complexes that transiently assemble with transcription factors and chromatin modifiers to regulate gene expression. In this study we analyzed data from 3,290 immuno-precipitations (IP) followed by mass spectrometry (MS) applied to human cell lines aimed at identifying CoRegs complexes. Using the semi-quantitative spectral counts, we scored binary protein-protein and domain-domain associations with several equations. Unlike previous applications, our methods scored prey-prey protein-protein interactions regardless of the baits used. We also predicted domain-domain interactions underlying predicted protein-protein interactions. The quality of predicted protein-protein and domain-domain interactions was evaluated using known binary interactions from the literature, whereas one protein-protein interaction, between STRN and CTTNBP2NL, was validated experimentally; and one domain-domain interaction, between the HEAT domain of PPP2R1A and the Pkinase domain of STK25, was validated using molecular docking simulations. The scoring schemes presented here recovered known, and predicted many new, complexes, protein-protein, and domain-domain interactions. The networks that resulted from the predictions are provided as a web-based interactive application at http://maayanlab.net/HT-IP-MS-2-PPI-DDI/.

## Author Summary

In response to various extracellular stimuli, protein complexes are transiently assembled within the nucleus of cells to regulate gene transcription in a context dependent manner. Here we analyzed data from 3,290 proteomics experiments that used as bait different member proteins from regulatory complexes with different antibodies. Such proteomics experiments attempt to characterize complex membership for other proteins that associate with bait proteins. However, the experiments are noisy and aggregation of the data from many pull-down experiments is computationally challenging. To this end we developed and evaluated several equations that score pair-wise interactions based on co-occurrence in different but related pull-down experiments. We compared and evaluated the scoring methods and combined them to recover known, and discover new, complexes and protein-protein interactions. We also applied the same equations to predict domain-domain interactions that might underlie the protein interactions and complex formation. As a proof of concept, we experimentally validated one predicted protein-protein interaction and one predicted domain-domain interaction using different methods. Such rich information about binary interactions between proteins and domains should advance our knowledge of transcriptional regulation by CoRegs in normal and diseased human cells.

## Introduction

CoRegs are members of multi-protein complexes transiently assembled for regulation of gene expression [1]. Assembly of these complexes is affected by ligands that bind to nuclear receptors (NRs), such as steroids, retinoids, and glucocorticoids [2]–[5]. CoRegs complexes exist in many combinations that are determined by post-translational modifications (PTMs) and presence of accessory proteins [6], [7]. To date, over 300 CoRegs have been characterized in mammalian cells [8] and it has been shown that CoRegs complexes control a multitude of cellular processes, including metabolism, cell growth, homeostasis and stress responses [6], [9], [10]. Many CoRegs complexes are considered master regulators of cell differentiation during embryonic and post-developmental stages [10], [11], and evidence suggests that malfunction of these proteins can lead to the pathogenesis of endocrine-related cancers [3], [12] and diabetes [13]. Importantly, it is believed that development of better chemical modulators of CoRegs will lead to a ‘new generation’ of drugs with higher efficacy and selectivity [14], [15].

To accelerate research in the area of CoRegs signaling, the Nuclear Receptor Signaling Atlas (NURSA) [16] have been applying systematic proteomic and genomic profiling related to CoRegs [17], [18]. Recently, the NURSA consortium released a massive high-throughput (HT) IP/MS study reporting results from 3,290 related sets of proteomics pull-down experiments [19]. The results from these experiments are protein identifications with semi-quantitative spectral count measurements, which can be used to approximate protein enrichment in individual IPs. Multiple IP experiments that sample different protein complex subunits can be integrated to gain a global picture of protein complex composition [20]–[22]. Several prior studies applied to human cells have proposed strategies to reconstruct protein complexes by combining results from HT-IP/MS [23]–[28]. Some of the results from such studies have been processed by algorithms that probabilistically predict binary protein-protein interactions (PPIs). In some cases, such predictions were validated using known PPIs from the literature, where in few cases predicted interactions were further validated experimentally. For example, Washburn and colleagues implemented the multidimensional protein identification technology (MudPIT) method to pull down complexes using 27 bait proteins from the Mediator complex to suggest 557 probabilistic interactions between the baits and their pulled preys [23]. They used the Jaccard distance to integrate protein co-occurrence in the different experiments, and compared their ‘high-confidence’ interactions with those listed in a literature-based database, the human protein reference database (HPRD) [29]. Experimentally, the study validated few predicted interactions using co-IP and western blots. In a follow up study, different clustering approaches to extract sub-complexes from related affinity purification (AP)-MS experiments using three distance measures: Manhattan, Euclidian, and Correlation Coefficient for clustering are described [30].

The aforementioned work, and other similar prior studies, ranked predicted associations and provided probabilities for interactions between baits and preys, building on the explicit nature of bait-prey relationship in epitope-based purifications. However, due to secondary cross-reacting proteins, bait-prey relationships are rarely explicit in IPs carried out with primary antibodies. Hence, here we developed and compared different ways, coded into mathematical functions, to score prey-prey interactions from a large, recently published, HT-IP/MS dataset. The equations predict direct protein-protein interactions between prey proteins without considering the specific baits. We also used the same equations to predict domain-domain interactions underlying the protein-protein interactions. We evaluated the performance of these equations using known protein-protein and domain-domain interactions from the literature and validated one protein-protein interaction experimentally, and one domain-domain interaction using computational docking. By combining the data from the 3,290 IP-MS experiments collected by NURSA we predicted binary interactions between prey proteins and their domains. We offer a global view of CoRegs complexes in human cells, and provide the predicted networks for exploration on the web through a web-based application with downloadable tables freely available at http://maayanlab.net/HT-IP-MS-2-PPI-DDI/.

## Methods

### IP-MS experiments

A detailed description of the IP-MS procedure can be found in references [19], [26] and the list of experiments in Dataset S1. The data we analyzed is provided as supporting material tables for reference [19]. These supporting tables contain GeneIDs for identified protein products, as well as the spectral count (SPC) measurements, and ‘abundance’ values, defined as SPCs/MW, where MW is the molecular weight for the largest isoform of the gene product. The latter normalization approximately accounts for the number of peptides expected from a protein. Abundance is logically similar to the normalized spectral abundance factor (NSAF) scores previously proposed [30], except the values are not scaled per experiment.

### Equations

To score prey-prey interactions from the HT-IP/MS data table, containing the ranks of proteins from the 3,290 IP-MS experiments, we evaluated existing and developed new equations implemented as algorithms in MATLAB and Java.

### Sørensen Similarity

Sørensen similarity coefficient (Sor) provides a symmetric similarity coefficient for comparing two finite sets. The coefficient ranges between 0 and 1, where 0 denotes no similarity, and 1 denotes identical sets. The Sørensen coefficient is calculated as the ratio of the cardinality of shared members between two sets and the sum of the cardinalities of the same sets.
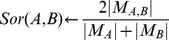
(1)The Sørenson coefficient was applied to determine the likelihood that proteins A and B directly interact. M_A_ and M_B_ are the sets of all experiments that reported either protein A, B or both as present in the lists of pulled prey proteins. M_A,B_ are lists where both A and B are present.

### Pearson's Correlation

Pearson's Correlation coefficient (Pr) characterizes the linear dependency of two variables. Here we used the Pearson's Correlation coefficient to quantify the correlation the SPC scores of two proteins across all IP/MS experiments.
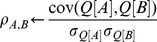
(2)ρ_A,B_ is the Pearson's Correlation coefficient between proteins A and B where Q denotes the reported ‘abundance’ which is SPC/MW (MW, molecular weight). 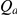 and 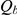 are the column vectors of Q at indices 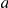 and 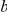. 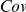 is the covariance and 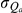 and 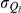 are the standard deviations of 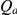 and 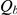.

### Equation 3

Equation 3 (E3) was developed through an intuitive manual symbolic search for functions that perform well, based on benchmarking, using known protein-protein interactions. E3 calculates a ratio between the sum of the SPC scores in experiment *j* (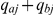) and the difference between the ranks of protein pairs based on their SPC scores in the same experiment. The average E3 scores across all experiments is the final score that is used to quantify the likelihood that two prey proteins interact. The rationale behind the E3 equation is to reward pairs of proteins that have similar SPC scores and similar ranks across all experiments, rewarding pairs of proteins with high SPC scores that appear in the same complexes.
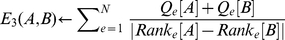
(3)


### AB Correlation

The AB correlation was also developed through an intuitive manual symbolic search for functions that perform well based on benchmarking using known protein-protein interactions. The AB correlation computes the mean of the product of SPC scores normalized by dividing by the sum of mean SPC scores across all experiments.
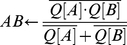
(4)The AB method also rewards pairs of proteins that have higher SPC scores in the same subset of experiments.

### PPIs from literature for validation

To evaluate the predicted prey-prey protein interactions using the four equations, we used an updated version of the human literature-based protein-protein interactome we developed for the program Genes2Networks [31]. The PPIs are from 12 databases: HPRD [29], MINT [32], DIP [33], MIPS [34], PDZBase [35], PPID [36], BIND [37], Reactome [38], BioGRID [39], SNAVI [40], Stelzl et al. [41], and Vidal and co-workers [42]. These databases contain direct physical interactions for mouse, rat, and human proteins containing 11,438 proteins connected through 84,047 interactions extracted manually from publications. We converted all IDs to human IDs using homologene (http://www.ncbi.nlm.nih.gov/homologene).

### Domain-domain interactions from the literature for validation

To identify domains for proteins, we used the Pfam domain database release 24.0. The file ‘Pfam-A.full.gz’ was downloaded from: ftp://ftp.sanger.ac.uk/pub/databases/Pfam/releases/Pfam24.0/on November 1st 2010.

Domain-domain interactions (DDI) were obtained from the Domine database [43]. The Domine database contains 26,219 domain-domain interactions. Among these domain-domain interactions, 6,634 were inferred from the protein data bank (PDB) and 21,620 were computationally predicted by one or more of 13 prediction methods. In order to score domain-domain interactions, we developed a prediction vector 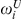 containing a combined score for all predicted PPIs that contain domain-pairs at each side of a scored PPI. We assigned the score of the predicted PPI to the DDI 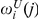 score.

### Western Blots and IPs to validate the interaction between STRN and CTTNBP2NL

Antibodies for STRN, also called Striatin, are polyclonal rabbit, and were purchased from Millipore Corp. Antibodies for CTTNBP2NL were purchased from GeneTex. MCF-7 cells were lysed in immunopreciptation buffer containing Hepes (50 mM, pH 7.4), NaCl (150 mM), EDTA (1 mM), Tween-20 (0.1%), glycerol (10%) and protease inhibitors. The lysates were pre-cleared in the presence of rabbit IgG and protein A beads. The input sample was collected after pre-clearing. Samples were rotated overnight with IgG or Striatin antibody and subsequently incubated for two hours with Protein-A beads. The washed protein-containing beads were denatured and analyzed by Western blot.

### Molecular dynamics simulations to validate interactions between the HEAT and PKinase domains of PPP2R1A and STK25

The MolSoft ICM software was used to perform the domain-domain docking simulation. ICM uses a two-step method: pseudo-Brownian rigid-body docking followed by biased probability Monte Carlo minimization of the ligand side-chains, to sample conformational space in order to identify the global energy minimum for a given interaction [44]. For this specific simulation, the protein PPP2R1A (PDB ID: 1B3U), the receptor, was kept rigid, while conformations of the ligand STK25 (PDB ID: 2XIK) were sampled around the receptor and corresponding docking scores were retrieved. Domains were then examined for interactions based on these scores.

## Results

We analyzed the experimental data from 3,290 IP-MS experiments targeting 1,083 antigens (bait proteins) using 1,796 different antibodies. These experiments detected 11,485 non-redundant proteins (Dataset S1). Some of the baits were pulled-down with several different antibodies. Some of the experiments with the same baits and antibodies were repeated several times but conducted under different conditions, i.e., stimulated/un-stimulated cells, or different cell types. Complexes are mostly isolated from nuclear fractions but some experiments use cytosolic fractions. Summary of the experimental conditions, cell types, antibodies and baits used, counts of normalized peptides identified in each experiment per protein, and size of the lists of proteins identified in each experiment can be directly obtained from the primary publication provided as reference [19].

IP-MS proteomics profiling have several known experimental challenges that need to be considered when applying functional global analyses on such data. First, it is well established that the proteins identified in such experiments are enriched for highly abundant and “sticky” proteins. This results in numerous proteins appearing frequently in almost all pull-downs regardless of the cell type, cellular fraction or experimental conditions. To address this we used a list of “non-specific” proteins to filter protein identifications that appear frequently in many pull-downs (Dataset S1). For all further analyses we removed these proteins from the results. Such a “non-specific” protein list can be useful as a guideline for filtering other IP-MS proteomics data applied to human cells. However, it should be noted that the concept of filtering IP-MS proteomics data based on a “non-specific” list is only meant as a guide. The sticky non-relevant proteins may play an important biological role that would be missed by removing them. In general, proteins that appear in the list are enriched in heat shock, ribosomal, and heterogeneous nuclear ribonucleoproteins (hnRNPs). Also, the majority of proteins on the non-specific list were selected based on the purifications from nuclear extracts, so some abundant cytosolic proteins may be over represented in the protein-protein and domain-domain interaction predictions since these may not have been removed. In order to integrate and visualize the results from the 3,290 IP-MS experiments, we first used the Jaccard Distance (JD) to construct a CoRegs complex similarity graph were nodes represent protein lists from each experiment and links represent overlap between experiments (Fig. S1). Nodes and links are preserved in the network if the similarity is greater than the Jaccard distance of 0.7. This retained 491 experiments and 2233 links between them, which are a small portion of all possible experiments and their similarities (Fig. S2A). On average, pull-down experiments reported the identification of ∼30–200 proteins but the distribution has a heavy tail with few experiments identifying over 1000 proteins (Fig. S2B).

**Figure 1:**
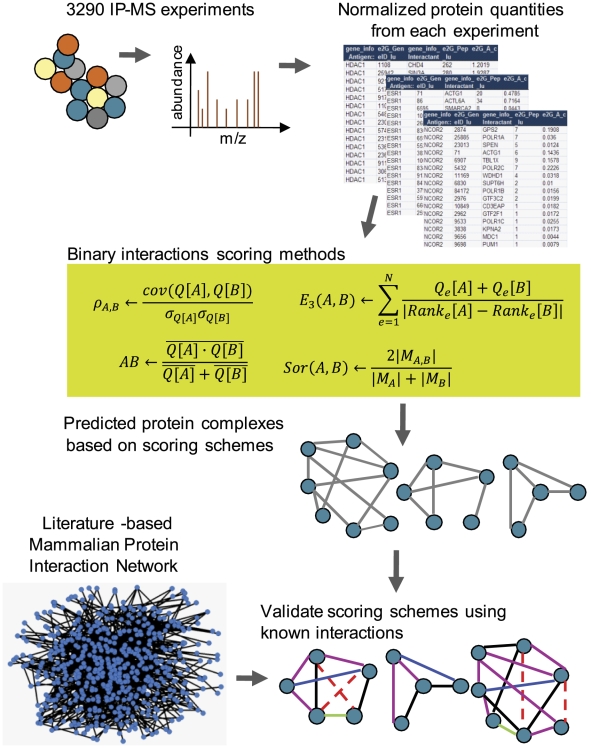
Workflow of the analysis of aggregated IP-MS experiments.

Our aim in this study is to assign confidence scores to binary prey-prey protein-protein and domain-domain interactions by integrating information from the 3,290 IP-MS experiments. The rationale for this approach is that the experiments, reporting lists of ∼30–200 proteins for each pull-down, taken together, provide enough information to reconstruct high-fidelity, small-sized complexes and potentially enough to recover direct physical interactions between pairs of proteins and domains. We reasoned that if we use all the information across all experiments to score each pair of proteins for potential direct interaction, we will be able to identify novel associations in addition to recovering known interactions better than by chance. In contrast with most prior methods that focused on scoring bait-prey interactions, our equations predict interactions between prey proteins that commonly reappear together in different pull-downs. Although the data collected for this study was aimed at the recovery of interactions between the intended antigens (baits) and other proteins, the majority of primary antibodies cross-react with multiple secondary antigens and those antigens interact with other proteins. This complicates bait-prey scoring of HT-IP/MS data. Yet, logically, if two proteins reappear together at the top of lists in many different pull-downs, we can guess that they may physically interact regardless of which baits were used to pull them down, making it possible to predict likely binary interactions by utilizing the spectral counts, not just co-occurrence. To encode such logic into mathematical functions we devised four scoring schemes, each attempting to address the problem in a slightly different way. To evaluate the performance of the four scoring schemes we used known PPIs we consolidated from online databases [31]. The overall schema for this approach is depicted in Fig. 1.

To compare the performance of the different scoring methods we visualized the results as either receiver operator curve (ROC) (Fig. S3), random walks (Fig. S4), or a sliding window (Fig. S5). Visualization of overlap between a ranked list and a gene set using a random walk was borrowed from the popular Gene-Set Enrichment Analysis method [45]. The three equations AB, E3, and Pr can be combined with the Sørenson coefficient to slightly improve the predictions by the AB and E3 equations, and significantly improve the predictions made with the Pr equation. AB and E3 perform best when combined with the Sørenson coefficient because these equations take into account the quantitative levels of the peptides, rewarding interactions that appear on top of the same pull-downs and penalizing potential interactions where the two proteins are not present in the same pull-down, or when one protein appears at the top and the other at the bottom. The different methods recover different sets of interactions and in some cases complement each other, suggesting perhaps that a combined weighted score may provide better results than using a single equation (Fig. S6, Dataset S2).

**Figure 2:**
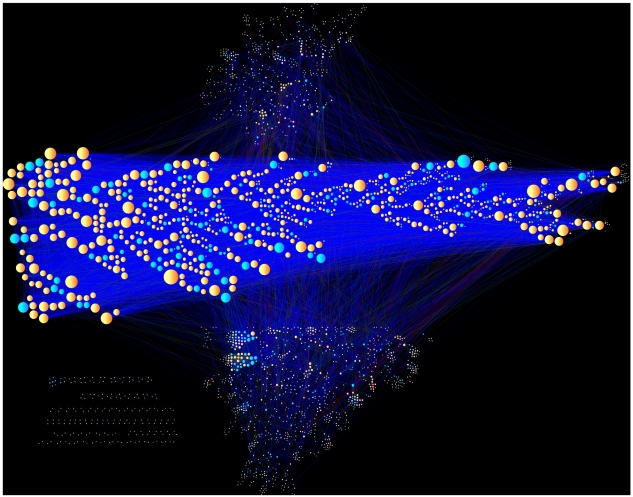
Network of predicted interactions comprised of 2509 proteins (nodes) and 28,886 interactions (edges) ranked by all three methods in the top 10% of predicted interactions. Yellow nodes are prey only and blue nodes were used as bait at least once. Edges are colored according to the following criteria: Blue edges are predicted interactions that do not have reported direct or indirect interaction in the literature; Green edges are predicted interactions that have one or more reported indirect interaction (one intermediate); Red edges are recalled direct interactions.

**Figure 3:**
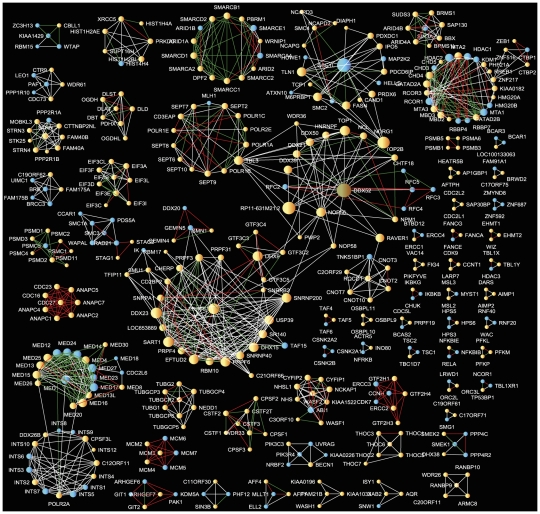
A network of predicted protein complexes containing 543 proteins and 1,893 interactions. Complexes are assembled by selecting and visualizing cliques formed by predicted protein-protein interactions ranked in the top 1% by all three methods. The resulting network composed of 63 protein complexes containing 3 to 25 proteins. Yellow nodes are prey and blue nodes are bait proteins. Edges are colored according by the following criteria: White edges are predicted interactions that do not have reported direct or indirect interaction in the literature; Green edges are predicted interactions that have one or more reported indirect interaction; Red edges are recalled direct interactions.

**Figure 4:**
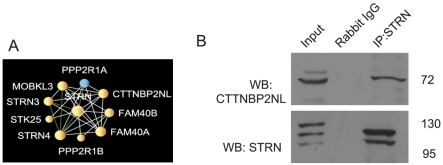
Confirmation of a binding interaction within the STRN complex. (A) Selected complex from Fig. 3 was further analyzed. (B) MCF-7 cells were lysed and STRN was immunoprecipitated. The species-matched immunoglobulin (rabbit IgG) was added to lysates in place of antibody as a negative control condition. The resulting immunoprecipitates were analyzed by Western blot for the presence of CTTNBP2NL (top panel). The blot was stripped and re-probed for STRN (lower panel).

Next, we used ball-and-stick diagrams to visualize the results across all experiments. We first visualized all overlapping interactions listed in the top 10% of predicted protein-protein interactions by each method (AB, E3 and Pr combined with Sor). This resulted in a network made of 2,509 proteins (nodes) and 28,886 interactions (edges) (Fig. 2). Using Cytoscape's organic visualization algorithm, the hubs of this network self-organize into an interesting hierarchical structure that may reflect their complex formation relationship. This network provides a global view of the CoRegs interactome, allowing zoom-in to view the identity of high confidence predicted protein-protein interactions and the complexes that these interactions form. To accomplish this zoom-in view, we increased the threshold to only include interactions from the top 1% of predicted interactions by all three scoring methods and include only three-node cliques. Three-node cliques are triangles in the network topology where three proteins are connected to each other with a maximum of three links. The resultant network contains 543 proteins and 1,893 interactions organized into 63 tightly connected protein complexes containing 3 to 25 proteins (Fig. 3). Many of the interactions and complexes that emerged are already known from low-throughput protein-protein interactions studies. However, some of the complexes within this network and many of the predicted protein interactions are novel. As a proof of concept, we focused on one predicted complex where most of the members of the complex were exclusively prey proteins in all experiments, and most interactions in the complex were not previously known (Fig. 4A). The complex contains ten densely connected proteins with the protein STRN in the center, predicted to interact with all other nine members. STRN, STRN3 and STRN4 are scaffolding proteins with a calmodulin binding domain. Interestingly CTTNBP2NL has been previously reported with STRN and STRN3 in another IP/MS study [46]. To experimentally validate one of the interactions within this complex we used IP and western blotting to demonstrate a direct interaction between STRN and CTTNBP2NL which is another member of the predicted complex (Fig. 4B). We chose this interaction based on antibody availability. Our experiment clearly shows that the two proteins interact. Such a demonstration of physical interaction experimentally does not prove that our prediction method works well, but it demonstrates how predicted interactions can be further validated experimentally. To prove that the predictions are of high quality, many such experiments need to be performed with appropriate controls to show statistically that the combined equations can predict, with high fidelity, physical interactions.

Before analyzing all of the 3,290 IP-MS experiments published by Malovannaya et al [19], we had access to a subset of the data before it was published. Therefore, we developed our analysis methods on a subset of 114 IP-MS experiments that are a fraction of the entire set of the 3,290 IP-MS experiments. In order to integrate and visualize the results from these 114 IP-MS experiments, similarly to the network shown in Fig. S1, we created the Jaccard Distance (JD) CoRegs complex similarity graph (Fig. S7). Most of these initial 114 experiments used Estrogen Receptor α (ESR1) and nuclear receptor co-activator 3 (NCOA3), also called SRC3, as baits in different cellular conditions. Both proteins play an important role in breast cancer, where SRC3 serves as the main co-activator of estradiol-dependent ESR1 [47], [48]. The experiments that used ESR1 and NCOA3 as baits resulted in similar protein lists (clusters in the subnetwork in Fig. S7) compared with the other pull-downs. Using the same prediction combined scores with the three equations, with lower thresholds, we identified five distinct high confidence complexes we named: SMARC, CSTF, RCOR, MBD, and SIN3A (Fig. S8). These five complexes have been previously reported in the Corum database [49] and some have been functionally characterized (Fig. S9). Specifically, the SMARC complex highly overlaps with complex IDs 238, 714, 803, and 806 in Corum, a database of reported protein complexes [49]. The CSTF complex is listed as complex number 1147 in Corum, RCOR is listed as 626, and MBD and SIN3A have associated IDs with highly overlapping entries for complexes in Corum. The SMARC and CSTF complexes were recovered mostly from ESR1 pull-down experiments, while the other three complexes are formed by combinations of many other types of baits. Notably, the SMARC and CSTF complexes are nearly mutually exclusive to two different antibodies targeting ESR1, and are recovered in the control experiment from HeLa cells that do not express ESR1. Thus, one antibody is likely cross-reacting with a member of the SMARC complex, whereas the other antibody cross-reacts with a member of the CSTF complex (Fig. S10). This result highlights the importance of protein complex reconstruction from HT-IP/MS based on prey-prey co-occurrence alone, independently of the intended baits.

Since PPIs are often the result of interactions between the structural domains of the interacting proteins, and since we know most of those domains for all pulled prey proteins based on their amino-acid sequences, we can use the scores for PPIs to also score and rank domain-domain interactions (DDIs). The scoring of domain interactions is slightly more complex since most proteins have several different domains and the domains can appear more than once within the same protein. To resolve this we used the score for PPIs containing domains between all possible domain pairs from each side of the PPI and normalized the score across all the domains (see methods). The aggregated score for all DDIs was accumulated across and within all 3,290 IP-MS experiments. The idea of predicting DDIs from PPIs is not new [50]–[52]. DDIs can also be predicted using structural biology methods or by evolutionary conservation of sequences across organisms [53]. To evaluate which PPI scoring method works best to predict DDIs, we compared the predicted scores for DDIs with reported DDIs from the Domine database. The Domine database contains both structurally observed and computationally predicted DDIs [43]. ROC curves and random-walk plots were used to evaluate DDI predictions, similarly to how we evaluated the PPI prediction methods (Fig. S11 and S12, Dataset S3).

**Figure 5:**
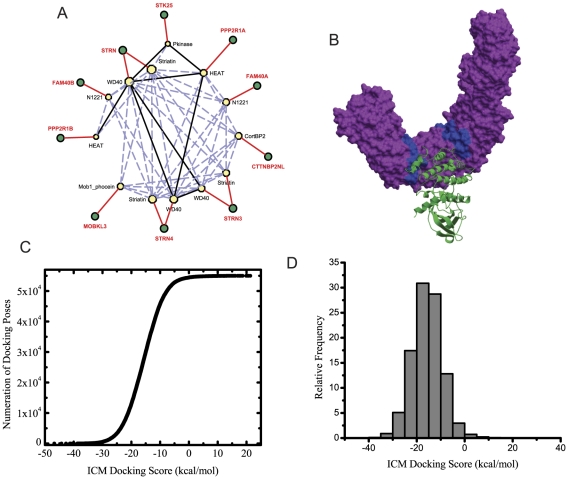
Validation of a domain-domain interaction. (A) Network showing the predicted DDIs for the predicted STRN protein complex. The network was constructed by importing domains for each protein from the PFAM database, associating protein domains to each of the proteins in the STRN complex, and using top predicted DDIs to connect the domains. In the network yellow octagons are domains and circles are proteins. Domains are connected to proteins using red, solid-black and dashed-blue edges. Black edges signify true positives and dashed-blue predicted DDIs. In the complex, PPIs that did not have a matching predicted DDI were eliminated. (B) Validation of a DDI interaction using molecular docking. The lowest energy conformation predicted by the docking simulations of STK25 to PPP2R1A. The interaction of the Pkinase domain with the HEAT domain is shown. (C) Binding energy landscape of all generated docking scores between STK25 and PPP2R1A. (D) Histogram of generated docking scores.

The plots show that we can reliably recover known and predicted DDIs. In addition to the four equations used to score PPIs, we introduced another scoring scheme, λ, for scoring DDIs. λ is an index that counts the number of times two predicted interacting prey proteins have a domain on each side of the PPI. Such an index improves DDI predictions. In addition to the type of analysis we did for PPIs, we also attempted to further combine different prediction methods to optimize DDI predictions. Finally we visualize our predicted DDIs with known DDIs as a network diagram to visually explore interactions among all domains (Fig. S13) and within the STRN centered complex identified by the PPIs predictions (Fig. 5A). To further validate one of the predicted DDIs we pursued a computational structural biology approach. We attempted to dock the PKinase domain of STK25 to the HEAT domain of PPP2R1A. We chose these two proteins because they had a crystal structure in PDB. Although the DDI is already listed in Domine, the prediction of this DDI interaction is based on sequence and homology. Hence there is no direct evidence of such interaction between these two proteins and their domains. Using the Molsoft ICM software we obtained a docking score of −46.75 kcal/mol. This score is considered high and as such confirms the interaction. By examining the confirmation of this interaction it appears that the Pkinase domain of the STK25 protein binds to the HEAT domain of PPP21RA. The energy gap of approximately 2 kcal/mol (ICM score units) between the best obtained and next consecutive docking score clearly suggests strong recognition of the HEAT domain by the Pkinase domain (Fig. 5B–D).

## Discussion

In this study we combined results from 3,290 experiments that identified nuclear protein complexes in human cells using IP-MS. We implemented and evaluated four different equations assessing their ability to predict direct physical PPIs from the aggregated proteomics data using known PPIs from the literature. The highest scoring predictions were visualized as networks with many densely connected clusters that are likely made of real protein complexes. The prediction scores for potential interactions could be considered as surrogates to real affinity constants. However, since we do not know the exact quantities of proteins, it is not possible to compute exact dissociation constants. Such binding constants can be useful for dynamical simulations where we could stochastically trace the transient dynamics of CoRegs complex formation in-silico. Scoring PPIs by only using the prey measurements may become more robust as more IP-MS experiments are published. However, careful attention should be given to weighting the repetitiveness of experiments so interactions from similar pull-downs, if repeated, are not mistakenly given higher scores. Regardless of possible limitations, the ability to recover direct PPIs based on such a massive dataset is an important step toward utilizing HT/IP-MS datasets for reconstructing networks and generating hypotheses. In addition, we show that the equations can be extended to predict interactions between structural domains. We also demonstrated two ways to further validate predicted PPIs and DDIs, using experimental and computational approaches. In summary, our analyses explored new methodologies for scoring PPIs and DDIs using data from related IP-MS experiments, providing many hypotheses about mammalian CoRegs complexes formation, and allowing users to explore novel complexes, PPIs and DDIs online at http://maayanlab.net/HT-IP-MS-2-PPI-DDI/. This resource can help us advance the catalogue of transcriptional regulation by CoRegs in normal and diseased mammalian cells.

## Supporting Information

Dataset S1 Information on each IP/MS experiment, quantity of proteins purified in each IP/MS experiment, size of protein lists purified in each IP/MS experiment, list of sticky proteins.
(XLSX)

Dataset S2 Scores for top 1% predicted PPIs by each method.
(XLS)

Dataset S3 Scores for top 1% predicted DDIs by each method.
(XLSX)

Figure S1 Each node in the network represents a list of proteins identified in one of the 3,290 IP-MS experiments color coded according to the bait protein targeted by an antibody in a single experiment. An edge represents the similarity between two lists using the Jaccard distance. A node is preserved if it has at least one edge with Jaccard distance <0.7. The network contains 491 nodes and 2233 edges. The diameter of a node represents the size of a list from a specific experiment.
(EPS)

Figure S2 (A) Histogram of Jaccard distances between pairs of 3,290 experiments. (B) Histogram of the size of pull-down lists from all IP-MS experiments.
(EPS)

Figure S3 (A) Receiver operator curve (ROC) of the recovery of known interactions using the different scoring methods. Recall rate of known PPIs (y-axis) is computed and displayed as a ratio between ranked predicted PPIs by each scoring method and known PPIs. (B) Area under the curve (AUC) computed for each method.
(EPS)

Figure S4 Running-sum of the top 1,563,309 predicted PPIs, predicted with the equations: (A) E3, (B) AB, and (C) Pr. The running-sum increases by √((u−t)/t) units if it encounters a known PPI, and decreases by √(t/(u−t)) units otherwise. The magenta line in each chart shows the walk when incorporating the Sørensen similarity. u and t are counts of predicted and known interactions in the current dataset respectively. The running-sum for a random sample of scrambled ranks of the same set of interactions along with the mean of running-sums of 1000 random samples are also included in each chart.
(EPS)

Figure S5 Moving average of a window of 2,000 ranks predicted PPIs visualized as a line graph. Sørensen similarity between pairs of proteins combined with other scoring schemas. The inset in each chart shows the recall for PPIs with evidence of indirect interaction, i.e., one intermediate. (A) E3, (B) AB, and (C) Pr.
(EPS)

Figure S6 (A) Venn diagram showing the overlaps between the three different scoring methods for the top 10% of predicted interactions. (B) Overlaps of known PPIs from predicted interactions represented in (Fig. 7A).
(EPS)

Figure S7 Similarity graph created from a subset of 114 IP-MS experiments. Nodes represent baits and links represent similarity using the Jaccard index. Nodes are colored based on the bait. Most experiments used Estrogen Receptor α (ESR1) and nuclear receptor co-activator 3 (NCOA3), also called SRC3, as baits under different conditions.
(EPS)

Figure S8 (A) Hierarchical clustering of the quantities of identified proteins from the subset of 114 experiments. Only proteins that were present in three or more IP experiments were included. (B) Network of predicted complexes. Complexes are formed by visualizing predicted protein-protein associations ranked in the top 1000 by all three scoring schemes. All nodes with connectivity of one were removed. Edges are colored according by the following criteria: Light blue are predicted interactions that do not have reported direct or indirect interaction in the literature; Green are predicted interactions that have one or more reported indirect interaction; Red edges are recalled direct interactions. Dotted gray edges are direct interactions which were not ranked in the selected range by the methods but are present in the literature. Nodes with a pink circle around them represent members of previously characterized complexes from the Corum database; Blue nodes represent proteins that were also used as baits it at least one of the experiments.
(EPS)

Figure S9 Heatmap of the percent overlap between the five complexes predicted from the subset of 114 experiments (columns) and complexes from the Curom database (rows).
(EPS)

Figure S10 Left: Hierarchical clustering of the quantities of identified proteins from the subset of 114 experiments (same as Fig. 12A). Right: Zooming into two clusters to visualize the segregation of two complexes pulled by two different antibodies targeting the same bait.
(EPS)

Figure S11 (A) Recall rate for previously reported DDIs from DOMINE (y-axis) as a function of the ratio of predicted DDIs ranked by one or a combination of the scoring schemes. (B) Area under the curve (AUC) for the ∼728 K ranked DDIs (left y-axis, dark bars) and AUC for the top 7 K predicted DDIs (right y-axis, light bars).
(EPS)

Figure S12 A comparative chart of running-sums, as described for Fig. 5, for the 728,632 predicted domain-domain interactions sorted based on the scores that have been calculated using three different methods: E3, AB, and Pearson's computed individually and combined with the Sørensen Similarity and λ; the chart also shows the running-sum for randomly shuffled ranks of the same set of predicted DDIs.
(EPS)

Figure S13 Network representation of the top 10% of predicted DDIs where nodes having 50 or more predicted interactions were removed for visualization clarity. The network contains 357 domains (octagons) and 773 edges (red and blue lines). Node sizes are proportional to their connectivity. Predicted and recalled DDIs are colored in light blue and red respectively.
(EPS)
